# Biomechanical Investigation of Bone Screw Head Design for Extracting Stripped Screw Heads: Integration of Mechanical Tests and Finite Element Analyses

**DOI:** 10.3390/ma16155470

**Published:** 2023-08-04

**Authors:** Kinda Khalaf, Arash Azhang, Chih-Hsiu Cheng, Mohammad Nikkhoo

**Affiliations:** 1 Department of Biomedical Engineering, Health Engineering Innovation Center, Khalifa University of Science and Technology, Abu Dhabi P.O. Box 127788, United Arab Emirates; 2 Department of Biomedical Engineering, Science and Research Branch, Islamic Azad University, Tehran 1477893855, Iran; 3 School of Physical Therapy and Graduate Institute of Rehabilitation Science, College of Medicine, Chang Gung University, Taoyuan 33302, Taiwan; 4 Bone and Joint Research Center, Chang Gung Memorial Hospital, Linkou 33305, Taiwan

**Keywords:** bone screw, screw head socket, screwdriver, stripped screw failure, finite element modeling

## Abstract

Enhancing the design of bone screw head sockets to prevent stripping and improve the torque required for smooth unscrewing is a significant challenge in orthopedic applications. This research aims to establish a quantitative methodology by integrating mechanical testing with finite element (FE) simulations to determine a safe limitation depth for the screwdriver when engaging with the hexagonal socket, thus avoiding stripped screw heads. A FE model was developed to investigate the biomechanical responses of the screw head design. Five custom-made hexagonal sockets were manufactured, and single load torsional tests were conducted to assess the mechanical performance of the screws and drivers. The results from the mechanical tests were compared with the FE simulations, demonstrating a close agreement and confirming the model’s validity. Furthermore, additional FE models were created to study the impact of manufacturing tolerances on the socket width and screwdriver width. The findings revealed that the maximum torque to failure for the four designs was lower than the margins specified in ISO 6475. Additionally, increasing the depth of the screwdriver led to higher maximum torque values. This research suggests that the technique of screw insertion, specifically the depth of the driver tool within the screw socket, holds greater importance in preventing stripped screw heads than the design and manufacturing width of the bone screw’s hexagonal socket and screwdriver. This confirms the importance of screwdriver engagement inside the bone screw socket to prevent stripped screw heads and sheds light on the added value of maximum torque prediction for future design modifications.

## 1. Introduction

Bone screws are the most frequently used implants in orthopedic procedures, whether they are used to properly secure fixation devices to bone or to fix the bone in place and promote its natural healing [1,2,3]. The typical structure of a bone screw includes a threaded region to restrain fractured bone fragments, as well as a head section to connect to other implants and transfer the applied torque [4]. For different screw types, a concave recess is generally designed to match with a screwdriver for insertion or removal during the surgical procedure [5]. However, slippage at the interface of the recess and screwdriver is one of the most commonly observed challenges in orthopedic applications [6]. Another critical factor that affects the mechanical function of bone screws is torquing (producing a load through a rotational force on a screw or bolt). Correct torque production is key to the successful application of bone screws, as improper torquing may lead to implant failure and/or tissue damage, thereby necessitating revision surgery and triggering further disability [7]. Revision surgery not only increases the risk of subjecting the patient to surgical complications but may also introduce further implant-associated challenges, such as fixation, interface, and biocompatibility; particularly, these challenges can arise because the average age of the population most likely to undergo orthopedic surgery is above 65 years of age [7,8]. These surgeries are also cost-intensive, which constitutes an economic burden on both patients and public health care systems [9]. Importantly, revisions may not always be feasible due to clinical constraints and/or health conditions, which results in permanent disability, where the retained implants may cause pain, infection, soft tissue irritation, and/or allergic/autoimmune reactions [10].

In current clinical practice, the approximation of the torque in non-locking screws (screws without a locking head system) continues to rely on the user’s intuitive assessment of the required force to attain optimal fixation [11,12]. Existing research on this subject highlights that, to date, many orthopedic practitioners perceive screw insertion as an ordinary and insignificant procedure, conducted subjectively without specific standards or comprehensive surgical protocols/training [13,14]. On the other hand, there is ample evidence in the literature that under-torquing can lead to screw loosening and unstable fixation [12], while over-torquing may cause thread stripping, cam-out failure or cold welding [7,15]; both under- and over-torquing can potentially result in an increased risk of implant failure and/or delayed bone healing. Furthermore, forcefully removing screws significantly increases the chance of stripping and causing severe damage at the socket and screwdriver interface, leading to additional clinical complications [10]. Difficult screw extractions also give rise to various challenges, such as prolonged operations, bleeding, infection, and potential neurovascular injuries [10]. These issues typically arise when screw head sockets are stripped, making the drivers ineffective. In such scenarios, clinicians resort to their experience and employ familiar techniques like screw head drilling, foil insertion, and reverse thread extraction, though the efficacy of these methods is not guaranteed [16].

Different head socket designs are currently employed for both orthopedic and dental screws, including hexagonal, Torx (star shape), and cross sketch configurations [17]. The hexagonal shape stands out as the most prevalent choice for the screw head socket due to its ability to optimize torque transfer conditions. This is achieved by enlarging the contact area and aligning the torque angle, resulting in a uniform distribution of stress. As a consequence, the hexagonal design enhances the overall performance of the screw, making it a popular and effective choice in various orthopedic applications [18,19]. Improving screw head socket designs to prevent stripping and to increase the torque to facilitate unscrewing remains an unresolved mechanical challenge [5,20]. An incompetently inserted torque may result in unstable fixation, while conversely, a higher insertion torque could lead to cam-out failure or cold welding [19,21]. While complementary instruments, including aiming sleeves, appropriate driver engagement, and torque-limiting devices, may minimize/prevent stripping, such solutions are not reliable and the outcomes are variable [1,22].

Experimental and clinical studies, using both in vitro and in vivo platforms, can provide relevant information regarding the biomechanical performance of bone screws. However, these studies are generally limited by their inability to estimate the detailed mechanical parameters within the structural components and typically only focus on clinical outcomes. Finite element (FE) modeling offers a potent, cost-effective, and practical approach for conducting non-invasive investigations related to diverse orthopedic implants [23]. By simulating complex mechanical interactions within the body, FE modeling allows researchers to assess the performance and safety of implants without the need for invasive procedures [3,24]. This technology facilitates a deeper understanding of implant behavior under different loading conditions, aiding in the design optimization and evaluation of orthopedic devices, ultimately contributing to improved patient outcomes and reduced development costs [23]. Numerous FE studies explored the use of bone screw-based implants for different clinical applications [24,25,26]. Nevertheless, there remains a gap in the literature regarding the influence of manufacturing tolerances in the augmentation of the conventional hexagonal socket design. Combining an adequate screwdriver depth with an optimal screw head socket and driver design is relevant for estimating the maximum torque-slippage value towards preventing screw slippage. Identifying the safe limitation depth of the screwdriver, while engaging with the hexagonal socket, holds significant importance in the design and manufacturing process to avoid stripped screw heads. The study hypothesized that by reducing misfit and improving compatibility between the socket and screwdriver components, bone screw constructs would exhibit greater resistance against rotational slippage. Additionally, the study anticipated that the impact of interfacial misfit would be more pronounced in sockets with shallower insertion depths. Therefore, the main objective of this study was to develop a quantitative methodology, based on the integration of mechanical testing with FE modeling, towards the investigation of bone screw head design for extracting stripped screw heads. The appropriate screwing technique as well as the association between the width of the hexagonal socket of the bone screw and screwdriver were also explored to avoid screw slippage.

## 2. Materials and Methods

**Figure 1:**
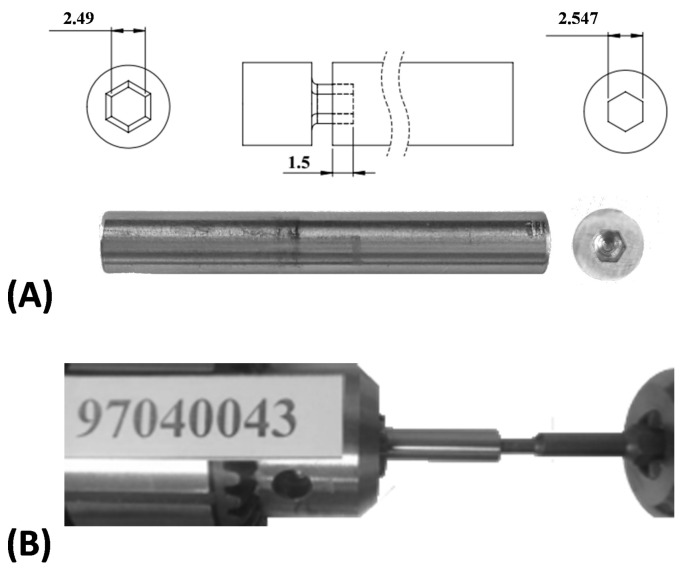
(**A**) A sample of manufactured socket and related dimensions, (**B**) torsional test using the Instron testing machine according to ASTM F543 and ASTM F116.

To perform the biomechanical experimental tests, five hexagonal custom-built screw heads and drivers were specially manufactured from a medical grade stainless steel rod (based on ASTM F 138 standard [27]). The CAD models of the socket and screwdriver (Figure 1A) were designed in Solidworks (Dassault Systemes, Vélizy-Villacoublay, France). A G-code was developed in PowerMill (Autodesk©, San Rafael, CA, USA) based on these CAD models to be used in the fabrication process. The yield stress of the stainless steel rod was 630 MPa, based on the reported data from the manufacturer (EZM EdelstahlZieherei Mark GmbH, Wetter, Germany). The sockets had a width of 2.547 mm and a depth of 1.5 mm (Figure 1A). Corresponding screwdrivers were manufactured and assembled in sockets for firm grip by a custom-made fixture (Figure 1A).

The mechanical performance of the sockets and screwdrivers was evaluated based on torsional tests using a mechanical testing machine (Instron Industrial Products, Norwood, MA, USA) according to ASTM F543 and ASTM F116 [28,29] (Figure 1B). A single load torque was applied using the servohydraulic mechanical testing machine. The sockets and screwdrivers were co-linearly mounted on the testing machine, and the alignment between the rotational centers of the screwdrivers and sockets was carefully verified before each mechanical test. Mechanical torsional loading was applied at a rate of 10 degrees per minute continuously until the measured torque significantly decreased and dropped to near zero. The values of the torques and angles were continuously recorded throughout the experimental test procedure, and the highest amount of torque was extracted for comparative study. The sufficiency of the number of specimens was evaluated by calculating the statistical confidence interval (CI) for the obtained data.

A nonlinear FE model, developed according to ASTM F543 and ASTM F116 testing techniques, was created to investigate the detailed mechanical responses of the screw head design observed in the experimental test. The implicit model was developed based on the aforementioned manufactured specifications of the socket and screwdriver using ABAQUS (SIMULIA, Providence, RI, USA) after applying torsional loading according to ASTM F543 and ASTM F116 criteria. The mechanical properties of the model were assumed as nonlinear, isotropic, elastic–plastic and were extracted from the experimental force–displacement data of the medical grade stainless steel rod. A plasticity model, based on the von-Mises yield criterion with hardening rule, was utilized in the simulations, while a large displacement technique with a total Lagrangian algorithm, was used for numerical calculations. The values of the elastic modulus and Poisson’s ratio were 195 GPa and 0.30, respectively. To generate the meshed FE model, 10-node quadratic tetrahedron elements were employed, and comprehensive sensitivity analyses were performed to assess the independence of the results from meshing size and numbers. The final number of elements and nodes used for the socket and screwdriver was 16,079 and 28,797, respectively.

The boundary conditions between the socket and screwdriver were determined using the discretization method of surface-to-surface with normal hard contact and tangential contact properties, which allows slippage and separation between the socket and screwdriver during numerical simulations. Coulomb’s tangential contact criterion with a friction coefficient equal to 0.3 was employed [30]; a torsional torque was applied, starting from zero to the maximum value obtained from experimental tests, with a ramp pattern to satisfy the general requirements of ASTM F543 and ASTM F116 criteria. The geometric nonlinearity was evaluated by the large deformation technique, while the Newton–Raphson force control methodology was used for analyses. The validity of the FE model was evaluated based on experimental tests.

Upon FE model validation, different models were developed towards studying the effect of the manufacturing tolerance of the socket width (SW) and screwdriver width (S). Initially, a critical tolerance for the 2.5 mm hexagonal socket and screwdriver was used to generate a model with a maximum socket width tolerance versus a minimum screwdriver width (i.e., SW = 2.547 and S = 2.47). Next, the critical tolerances were changed while selecting a bigger SW variation as a potential scenario in manufacturing. Considering four different inserted screwdriver depths, 16 different design modes were selected for comparison using the FE analysis as depicted in Table 1.

**Figure 2:**
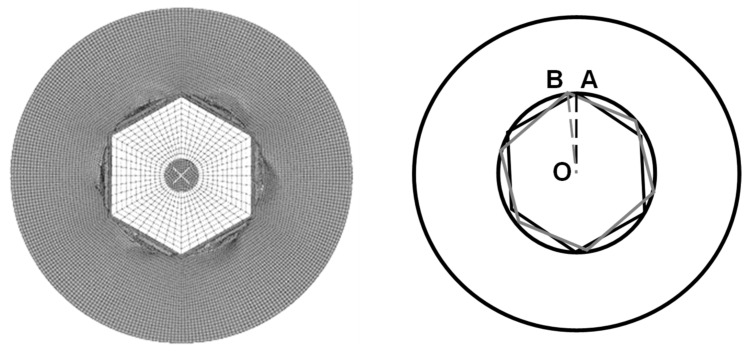
Slippage-resisting length definition (i.e., the length difference between OA and OB).

The 16 FE models with different geometries were then simulated under the torsional loading and boundary conditions previously discussed. The results of maximum torque and stress distribution were analyzed and compared to investigate the influence of screw head design for extracting stripped screw heads. In addition, the slippage-resisting free rotation angles were extracted as an important index to investigate the variations in the ability of resisting cam-out failure for different groups in this study. For this purpose, this geometrical angle was defined as the angle between the lines OA and OB, in which the point O is the center, point A shows the initial contact point, and point B shows the deformed position of point A at the end of simulation (Figure 2).

## 3. Results

**Figure 3:**
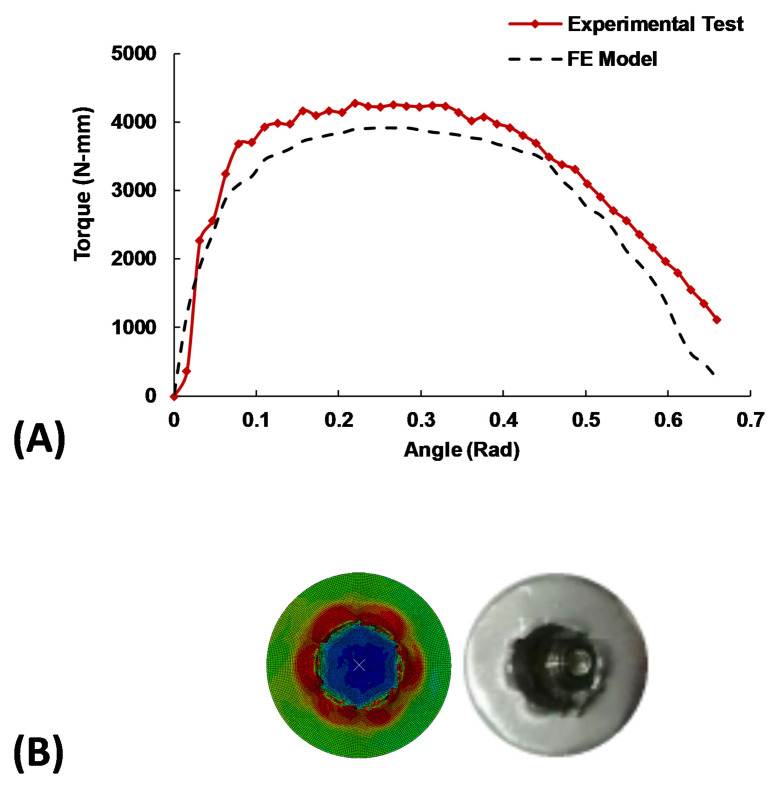
(**A**) Comparison of the experimental data for sample No. 1 and FE results. (**B**) Comparison of the simulation and the test result after slippage.

The average maximum torque measured in the experimental tests based on the five samples was 4097.4 (±118.15) N·mm, as compared to the relevant calculated torque of 3919 N·mm obtained from the FE model. Overall, the FE model results were well comparable with the experimental tests (Table 2), confirming the validity of the model (Figure 3). The statistical analysis based on calculating the CI confirmed that the sample size was sufficient with a confidence level of 95%.

**Figure 4:**
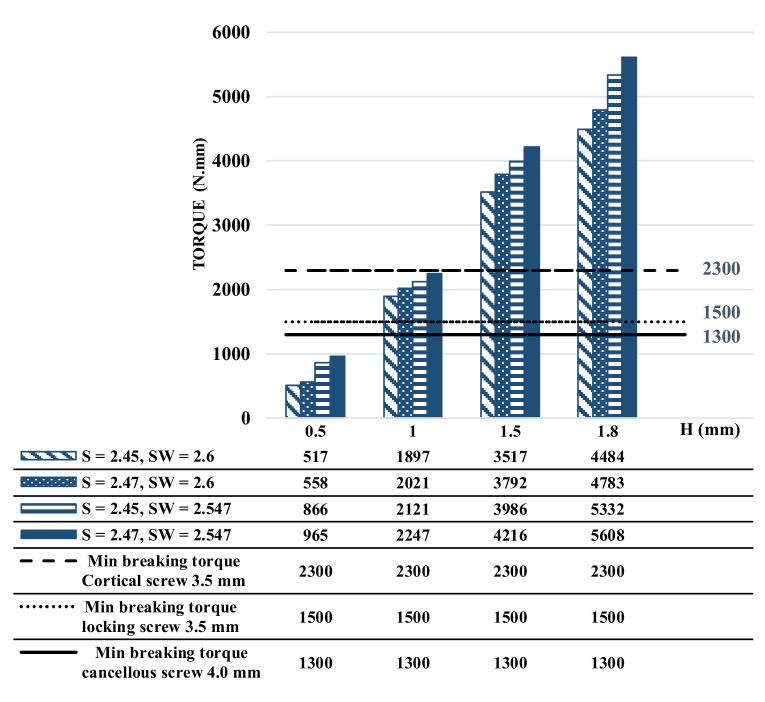
Comparison of the calculated maximum torque based on different screw head socket and screwdriver design parameters.

The simulations for 16 different conditions indicated that the maximum torques to failure for the four designs with the lowest insertion depth (i.e., 0.5 mm) were less than 1000 N·mm, which is less than the margins specified in ISO 6475. By increasing the insertion depth from 0.5 mm to 1 mm, the maximum torque increased on average by 108.52%, and the increase was 353.58% and 442.63% for 1.5 and 1.8 mm insertions, respectively (Figure 4). The results also demonstrated a similar trend of increase in the maximum torque associated with changing the manufacturing tolerances (Figure 4).

**Figure 5:**
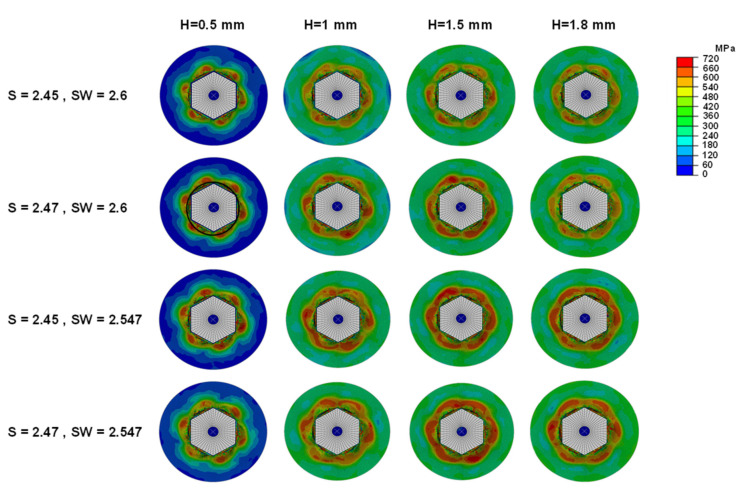
Comparison of the calculated von-Mises stress distribution on different screw head socket and screwdriver design parameters.

The von-Mises stress distributions of the maximum torque in the sockets are shown in Figure 5 for 16 different FE simulations. The results indicate that the stress patterns of the four sockets with the lowest insertion depth (i.e., 0.5 mm) were quite different from the other conditions. Generally, decreasing the SW values from 2.6 to 2.547 mm resulted in increasing the stress in the sockets with the same S values. Similarly, increasing the S values from 2.45 to 2.47 mm increased the stress in the sockets with the same SW values (Figure 5).

**Figure 6:**
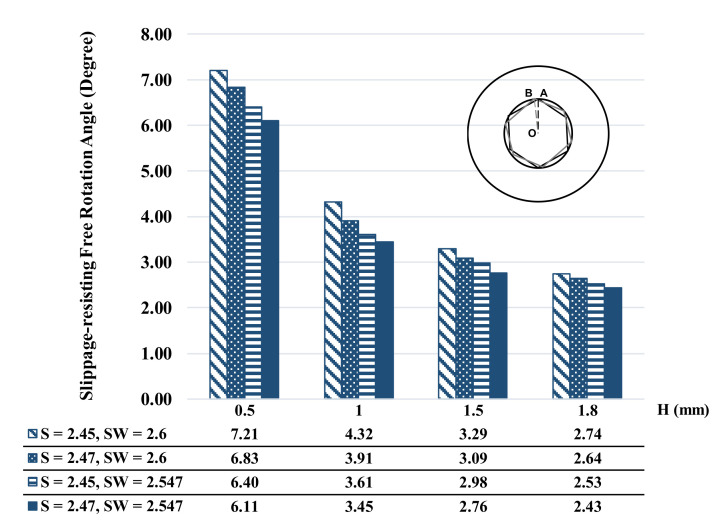
Comparison of the calculated slippage-resisting free rotation angle on different screw head socket and screwdriver design parameters.

For different FE simulation results, slippage-resisting free rotation angles were presented in Figure 5. Changing the manufacturing tolerance showed the same variation patterns in different inserting-depth groups. Decreasing the S values or increasing the SW values resulted in higher slippage-resisting free rotation (Figure 6). Increasing the insertion depth from 0.5 to 1 mm decreased the free rotation index by 6.12 (±2.46) % on average. These variations were 11.19 (±3.79) % and 15.70 (±3.62) % for increasing the insertion depth from 0.5 to 1.5 mm and 1.8 mm, respectively.

## 4. Discussion

Although the options for operative fracture treatment using metal implants have substantially increased in the last decade, orthopedic surgical implant removal remains challenging and riddled with complications [5]. This is particularly relevant during the removal of bone screws, as surgical difficulties can occur when a screwdriver slips through the screw recess once the twisting torque exceeds a certain level. Some of the factors that lead to such failure include the design, manufacturing, and/or material of the screw and the screwdriver [20,22]. Importantly, the design of the screw and screwdriver are key factors contributing to clinical complications during implant removal. The recess in the screw head, commonly known as the screw slot or screwdriver slot, is an essential component which allows the screwdriver to engage with the screw for tightening or loosening purposes. However, a non-optimal design of the screw slot may increase the likelihood of slippage during removal [19].

While efforts have been made by manufacturers to improve the design of screw heads (introducing different shapes, such as hexagonal, star, and cross heads, to enhance the engagement between the screwdriver and the screw [6,17]), slippage continues to be a key challenge leading to various complications during orthopedic removal procedures. Apart from design, the manufacturing process of the screws and screwdrivers can also influence the success of implant removal. Non-precise tolerances during manufacturing lead to a poor fit between the screwdriver and the screw recess [6,20]. This may result in instabilities and an increased risk of slippage during the removal process.

This study aims to provide a valid quantitative methodology towards the investigation of bone screw head design for extracting stripped screw heads. Custom-made sockets were designed and manufactured, and single load torsional tests, using a standard mechanical testing machine, were conducted to evaluate the performance of the screws and drivers. The obtained results from the experimental tests were then compared with a nonlinear FE model specifically developed and validated for this purpose. The FE model was created to provide a comprehensive understanding of the intricate biomechanical behavior of the screw head design, based on the standard testing techniques outlined in ASTM F543 and ASTM F116. To ensure the model’s accuracy and reliability, a rigorous validation procedure was employed, comparing the model’s predictions with experimental data from five distinct samples. The validation process yielded promising results, demonstrating an acceptable error rate of less than 15% for all five samples (Table 2 and Figure 3). These outcomes confirmed the FE model’s validity and robustness in accurately simulating socket behavior. However, the FE simulations’ slight underestimation compared to the experiment is likely due to computational simplifications in the material and boundary condition definitions. Notably, the study’s findings align well with reported results in the existing literature [20].

Different FE models were consequently developed to investigate the impact of the socket width (SW) and screwdriver width (S) on manufacturing tolerances in relation to screw head design for extracting stripped screw heads. Four different depth insertions (i.e., 0.5 mm, 1 mm, 1.5 mm, and 1.8 mm) were simulated by way of emulating real-life clinical surgical scenarios. It is noteworthy that during the process of bone healing, it is common for the socket to be filled with ingrown tissue, which can create a challenge where the screwdriver is unable to fully penetrate the socket’s depth. This condition can affect the stability and functionality of the bone screw. On the other hand, the findings of this study reveal that the impact of screwdriver depth on the maximum torque is more significant than variations in the width of the hexagonal socket (SW) and screwdriver (S). This suggests that adjusting the depth of the screwdriver has a greater influence on the maximum torque than modifying the dimensions of the hexagonal socket and screwdriver width. Therefore, it is recommended to ensure an appropriate depth of the screwdriver as a critical factor for achieving optimal torque and implant stability, even in cases where ingrown tissue might impede the complete insertion of the screwdriver into the socket. Highlighting the relative importance of screwdriver depth over SW and S width variations provides valuable insight in terms of considering the depth factor during surgical procedures and implant placement to optimize the biomechanical performance and long-term success of the implant. The effect of socket shape is another important factor [19] which was not the aim of the current study. We only explored the hexagonal socket shape here, as it is the most common design for screw heads [6,17]. Further work is recommended to explore innovative tools and techniques towards overcoming the challenges posed by ingrown tissue, ensuring effective engagement between the screwdriver and the socket and, ultimately, enhancing the overall outcome of orthopedic procedures.

In accordance with ISO 6475, the minimum breaking torque values are established for different types of screws based on the nominal thread diameter [31]. For example, for 3.5 mm nominal thread diameter cortical screws, the minimum breaking torque is estimated at 2300 N·mm. Similarly, for 4.0 mm nominal threaded diameter cancellous screws, the minimum breaking torque is 1300 N·mm, while for 3.5 mm nominal threaded diameter locking screws, it is 1500 N·mm [31]. The FE simulations in this study showed that the maximum torque to failure for some cases was lower than the standard values specified by ISO 6475 (Figure 4). This implies that the screws tested in these specific scenarios failed to withstand the minimum breaking torque established by ISO standards. Importantly, these failures were principally associated with the insertion depth of the screwdriver into the socket. The FE simulation results indicated that when the screwdriver was not inserted deeply enough into the socket, the maximum torque to slippage decreased. This decrease in torque capacity can be attributed to the reduced engagement between the screwdriver and the socket, leading to a weaker connection and potential failure. These results emphasize the significance of ensuring adequate insertion depth of the screwdriver during implantation procedures, as well as the critical role of proper technique and precision in achieving optimal engagement and torque transmission between the screw and the socket. By adhering to recommended insertion depths, surgeons can enhance the overall stability and longevity of the implant, reducing the risk of failure associated with insufficient torque capacity. These efforts may help align the results of this study with the standard breaking torque values specified by ISO 6475, ensuring the reliability and safety of orthopedic implant systems.

In socket–screwdriver constructs, interfacial misfit is an inherent consequence of manufacturing tolerances and design parameters [20,32]. The misfit refers to the variation or mismatch between the socket and screwdriver components, which can affect the performance and stability of the implant. To evaluate the actual points of initial contact at the socket–screwdriver interfaces, the slippage-resisting free rotation angle was defined in this study as a comparative index. Determining this angle allows for the evaluation of the resistance to rotational slippage and provides insight into the effectiveness of the interface. In this study, we additionally investigated the influence of manufacturing tolerances by analyzing the slippage-resisting free rotation angle across different groups with varying insertion depths. Our findings revealed consistent patterns in the variation of slippage-resisting free rotation angles when manufacturing tolerances were changed. Specifically, reducing the S values (indicating improved manufacturing precision) or increasing the SW values (reflecting a wider screwdriver) resulted in higher slippage-resisting free rotation angles (as depicted in Figure 6). This suggests that reducing misfit and increasing compatibility between the socket and screwdriver components lead to improved resistance against rotational slippage. Importantly, this study also highlighted that the impact of interfacial misfit is more pronounced in sockets with lower insertion depths. This suggests that when the screwdriver has limited penetration into the socket, even a slight misfit may affect the stability of the construct. Consequently, ensuring optimal manufacturing tolerances and design parameters becomes particularly crucial for sockets with shallow insertion depths to mitigate the risk of rotational slippage and enhance overall implant stability [6]. The significance of interfacial misfit and its impact on the performance of socket–screwdriver constructs are highlighted by these findings. Understanding the correlation between misfit, insertion depth, and slippage-resisting free rotation angles enables researchers and manufacturers to enhance design parameters and manufacturing processes, thus improving the reliability and effectiveness of orthopedic screws. This knowledge empowers them to optimize implant technology and advance patient outcomes [33].

Certain limitations in the current study should be deliberated here. The first is related to modeling the socket and screwdriver while neglecting the complete bone fixation system, including the bone tissue. This simplification was adopted to focus on the main objective of this study and reduce the computational cost, although future work can benefit from a more inclusive model. The second limitation lies in applying pure torsional torque without consideration of compressive loading during implantation. Although this is a common simplification in related studies in which experimental protocols are designed based on the aforementioned standards, a supplementary study to evaluate the results for combined loading would enhance the findings; it could also be coupled with the current FE methodology for future work. Furthermore, the ultimate failure torque may be slightly higher than the highest achievable torque during manual use for ordinary screwdrivers in surgery. However, the results achieved in this investigation can be regarded as a valuable guideline for approximating the maximum torque in bone screw applications. The outcomes of this work provide insight into the biomechanical interaction of the socket and screwdriver engagement and may serve as a basis for informing future design modifications aimed at optimizing their performance. By utilizing the results as a reference point, researchers and designers can assess the potential impact of various modifications to bone screw heads on their torque-bearing capacity, including improved screw head designs with enhanced strength and stability.

## 5. Conclusions

Despite the common use of bone screws and the frequently associated complications in orthopedic implant surgeries, there remains a gap in the literature regarding the influence of manufacturing tolerances for the augmentation of the conventional hexagonal socket design. The main contribution of this work lies in providing a modular FE-based methodology for identifying the safe limitation depth of the screwdriver while engaging with the hexagonal socket to avoid stripped screw heads. This study also emphasizes the relevance of the combination of an adequate depth of the screwdriver with optimal screw head socket and driver designs towards estimating the maximum torque-slippage value and preventing screw slippage. The results confirm that the screwing technique, or depth of the driver tool inside the screw socket, is more important than the design and manufacturing width of the bone screw hexagonal socket and screwdriver. This work also emphasizes the importance of screwdriver engagement inside the bone screw socket, while shedding light on the value of maximum torque prediction for future design modifications. Finally, understanding the relationship between misfit, insertion depth, and slippage-resisting free rotation angles allows researchers and manufacturers alike to refine design configurations, parameters and processes towards enhanced safety, reliability, and effectiveness of orthopedic screws.

## Figures and Tables

**Table 1:** Different design modes for FE analyses.

	Group 1		Group 2		Group 3		Group 4	
	H1 = 0.5 mm		H2 = 1.0 mm		H3 = 1.5 mm		H4 = 1.8 mm	
Row	SW	S	SW	S	SW	S	SW	S
1	2.6	2.45	2.6	2.45	2.6	2.45	2.6	2.45
2	2.6	2.47	2.6	2.47	2.6	2.47	2.6	2.47
3	2.547	2.45	2.547	2.45	2.547	2.45	2.547	2.45
4	2.547	2.47	2.547	2.47	2.547	2.47	2.547	2.47
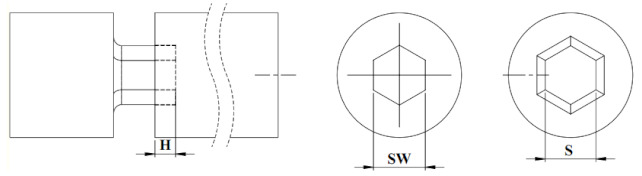								

H: Inserted depth of the screwdriver, SW: Socket width, S: Screwdriver width.

**Table 2:** Comparing torque to slippage for five experimental tests versus the two FEM analyses.

Sample No.	Test Report (N·mm)	FEM (N·mm)
No. 1	4253	3919
No. 2	4129	
No. 3	4099	
No. 4	3923	
No. 5	4083	
Average (Standard Deviation)	4097.4 (±118.15)	

## Data Availability

The data of the present work is available on demand from the corresponding author.
